# Fundamental Study on the Impact of Gluten-Free Starches on the Quality of Gluten-Free Model Breads

**DOI:** 10.3390/foods5020030

**Published:** 2016-04-21

**Authors:** Stefan W. Horstmann, Markus C. E. Belz, Mareile Heitmann, Emanuele Zannini, Elke K. Arendt

**Affiliations:** School of Food and Nutritional Sciences, University College Cork, College Road, Cork, Ireland; s.horstmann@umail.ucc.ie (S.W.H.); M.Belz@bfreefoods.com (M.C.E.B.); m.heitmann@umail.ucc.ie (M.H.); e.zannini@ucc.ie (E.Z.)

**Keywords:** coeliac disease, gluten-free, scanning electron microscopy, rapid visco analyser, lipids, crumb firmness

## Abstract

Starch is widely used as an ingredient and significantly contributes to texture, appearance, and overall acceptability of cereal based foods, playing an important role due to its ability to form a matrix, entrapping air bubbles. A detailed characterisation of five gluten-free starches (corn, wheat, rice, tapioca, potato) was performed in this study. In addition, the influence of these starches, with different compositional and morphological properties, was evaluated on a simple gluten-free model bread system. The morphological characterisation, evaluated using scanning electron microscopy, revealed some similarities among the starches, which could be linked to the baking performance of the breads. Moreover, the lipid content, though representing one of the minor components in starch, was found to have an influence on pasting, bread making, and staling. Quality differences in cereal root and tuber starch based breads were observed. However, under the baking conditions used, gluten-free rendered wheat starch performed best, followed by potato starch, in terms of loaf volume and cell structure. Tapioca starch and rice starch based breads were not further analysed, due to an inferior baking performance. This is the first study to evaluate gluten-free starch on a simple model bread system.

## 1. Introduction

Starch is the primary energy storage compound in many plants including cereals, legumes, potatoes and tubers and provides 70%–80% of the calories consumed by humans worldwide [1]. Starches are widely used as ingredients in many foods to improve appearance, texture, and overall acceptability. They are used as gelling, thickening, adhesion, moisture-retention, stabilizing, film forming, and texturizing agents [2]. In gluten-free products, starch may be successfully incorporated into the food formula to improve one or more of these properties depending on the interaction with other ingredients in the formulation and the type of food products.

The main starch sources in gluten-free systems are corn, tapioca, potato, rice, and gluten-free wheat [3]. Native starch exists in the form of granules. The size, shape, and molecular arrangement inside the granules depend on the species, cultivar, variety of plant, as well as the environmental growing conditions. The starch biosynthesis pathway generally results in two types of glucose based polymers being formed, the semi-linear amylose and the highly branched amylopectin. In addition, other components like proteins and lipids are associated with starch [4,5]. Cereal starches are usually considered gelling materials, and in baking they significantly contribute to texture, appearance, and overall acceptability of cereal based foods [4,5]. During the bread baking process, starch granules gelatinize, *i.e.*, they swell and are partially solubilised, but still maintain their granular identity [6]. Starch gelatinisation plays an important role in gluten-free formulations, due to the ability of starch to form a matrix in which air bubbles are entrapped. For this reason, the addition of gel forming starches such as pregelatinized starches and air cell stabiliser such as gums have been suggested as a means to provide gas occlusion and stabilizing mechanisms [2]. Moreover, the addition of a certain type of starch in the gluten-free formula could improve the batter consistency during mixing, enhance the softness of the crumb and control starch gelatinization during the baking process [7].

Gluten-free products are important for people who suffer from coeliac disease (CD), an immune mediated enteropathy causing inflammation in the small intestine [8]. It is triggered by the ingestion of prolamins from wheat, rye, and barley in genetically susceptible individuals [8]. CD is one of the most widespread food intolerances with a prevalence of 1% [8]. The avoidance of gluten intake is currently the only safe treatment for coeliac disease. This means that patients with CD have to strictly adhere to a gluten-free diet, being unable to enjoy common foods such as bread, pizza, pasta, or beer that are commonly based on gluten-containing grains. Due to the increasing prevalence of CD, there is a growing demand for palatable and nutritious gluten-free products from consumers. Hence, scientists aim to develop gluten-free products, which include a complex and well-matched list of ingredients to guarantee the production of high quality gluten-free breads with improved health and nutritional properties [9]. Recent studies have demonstrated a number of options for gluten replacement, including the use of various combinations of hydrocolloids [10], modifying the interaction between gluten-free proteins and starches [11], and the use of pseudo cereal flours such as quinoa [12]. In addition, the impact of modified starches on gluten-free dough and bread has been investigated by Ziobro *et al*. [13]. Modification of starch has an important impact on the consistent production of good quality gluten-free products. There are many commercial gluten-free breads on the market using an array of ingredients, without knowing how these ingredients interact and influence final product quality.

The present work is novel in its use of a simple gluten-free model formulation (seven ingredients), enabling the examination of the bread making performance of different starches. It aims to deepen the knowledge on how different starches influence baking performance. This is accompanied by a wide range of analyses characterising the gluten-free starches.

## 2. Materials and Methods

### 2.1. Materials

The suppliers for the ingredients used were Cargill, UK for corn starch; Agrana, Austria for potato starch; Roquette, France for gluten-free wheat starch; Tradelink, UK for tapioca starch and Beneo Remy, Belgium for rice starch. Dry yeast was supplied by Puratos, Belgium; sugar from Siúcra Nordzucker, Ireland; salt from Glacia British Salt Limited, UK and hydroxypropyl methylcellulose (HPMC) from J. Rettenmaier, Germany.

### 2.2. Microscopy

Samples of starch were dried in an air-oven for 1 h at 103 °C. Samples were affixed with double-sided carbon tape to an aluminium stub and coated with a layer of 25 nm of sputtered palladium-gold. Hereupon, samples were examined under high vacuum in a field emission scanning electron microscope (SEM) with a working distance of 8 mm. Secondary electron images were acquired at an accelerating voltage of 5 kV. For processing of the images SEM Control User Interface software, Version 5.21 (JEOL Technics Ltd., Tokyo, Japan) was used.

### 2.3. Particle Size

Analysis of the particle size distribution was carried out by laser diffraction using a dry feed cell (Malvern Mastersizer 3000, Instruments Ltd., Worcestershire, UK). The sample was dispersed in air using 1.5 bar pressure and measured when an obscuration of 5%–7% was achieved, with a refractive index of 1.45.

### 2.4. Chemical Characterisation of the Starches

Chemical characterisation of the starch samples was performed using laboratory standard methodologies. Moisture determination was performed using the air oven method (AACC Method 44-15 A). Total starch (AACC Method 76.13), damaged starch (AACC Method 76-31.01), amylose/amylopectin content of total starch, α-amylase activity (AACC Method 22-02.01) and β-amylase activity were determined utilizing commercially available assay kits (Megazyme International, Ireland Ltd., Wicklow, Ireland). The protein content was determined using the Bradford assay with bovine serum albumin as the calibration standard. For the determination of the lipid content, the starch samples were first digested according to the Weibull-Stoll method, to release bound lipids. The lipid content was then determined using AACC Method 30-25.01.

### 2.5. Rapid Visco Analysis

The pasting properties of the starch samples were determined according to the Newport Scientific Method 6, Version 4, December 1997, using a RVA Super 3 Rapid Visco Analyser (Newport Scientific, Warriewood, Australia) with thermocline control and data collecting software. Starch sample of 3 g (on a basis of 14% moisture) were weighed into an RVA aluminium canister, and 25 g of distilled water were added, to prepare a starch-water sample of 9.2% (*w*/w*w*). The temperature profile used was heating to 95 °C (6.3 °C/s), holding at 95 °C for 162 s and cooling to 50 °C (5.1 °C/s) and holding at 50 °C for 120 s.

### 2.6. Bread Making Procedure

Bread samples were produced based on a simple recipe (100% starch, 80% water, 2% HPMC, 2% salt, 4% sugar, 2% yeast, based on starch weight). For the pre-fermentation, yeast was suspended in warm water (25 °C) and regenerated for a period of 10 min. Mixing was carried out with a k-beater (Kenwood, Havant, UK) at low disk speed (level 1 of 3) for 1 min in a Kenwood Major Titanium KM 020 Mixer (Kenwood, Havant, UK). After that the dough was scraped down from the bowl walls and a further mixing of 2 min at higher disk speed (level 2 of 3) was carried out. The batter was scaled to 300 g into nine baking tins of 16.5 cm × 11 cm × 7 cm and placed in a proofer for 45 min at 30 °C and 85% relatively humidity (RH). The breads were baked for 55 min at 220 °C top and bottom heat in a deck oven, previously steamed with 0.7 L of water. After baking, bread loaves were removed from the tins and cooled at room temperature for 2 h. The loaves were subsequently analysed or packaged in plastic bags (polystrol-ethylene vinyl alcohol-polyethylene) for storage. Each starch bread batch was prepared in three replicates.

### 2.7. Bread Characteristics

The bread analyses were performed 2 h after baking. Four of eight loaves were immediately used for performing the texture and structural analyses on day 0, the remaining four loaves were used for the texture analysis on storage day 2 (50 h after baking) and storage day 5 (122 h after baking), two each day. For the analysis performed on day 2 and 5, the bread samples were packaged in polythene bags (polystyrol-ethylene vinyl alcohol-polyethylene) and stored at room temperature. Specific volume was determined using a Vol-scan profiler (Stable Micro Systems, Godalming, UK), equipped with a non-contact measurement device. The bake loss was measured as the difference between the initial weight of the sample (dough in the tin before fermentation) and the final weight of the sample (bread after baking and cooled for 2 h). For measuring the moisture of the bread crumb an air oven was used according to AACC Method 44-15A. The texture profile analysis (TPA) of the bread samples was determined using a universal testing machine TA-XT2i (Stable Micro Systems, Godalming, UK). This measurement, also called the “two-bite-test”, evaluated seven parameters assigning independent numeric values to bread attributes normally estimated with sensory test. For analysing the texture changes over times, the measurement was done on day 0 (the baking day), day 2, and day 5. On the baking day, two breads were sliced transversely by using a slice regulator and bread knife to retain consistent slices of 20 mm thickness. Eight bread slices, taken from the center of the breads, were used to evaluate the texture parameters using a universal testing machine TA-XT2i equipped with a 25 kg loading cell and a 35 mm aluminium cylindrical probe. The settings used were a test speed of 5 mm/s with a trigger force of 25 g to compress the middle of the bread crumb to 50% of its original height. The measurement was repeated after 2 and 5 days of storage using the bread samples packaged in a plastic bag and stored at room temperature. For crumb grain evaluation a C-cell Bread Imaging system (Calibre Control International Ltd., Warrington, UK) equipped with C-Cell version 2.0 software was used. The area of holes (the total area of holes as a percentage) and the wall thickness (the average thickness of cell walls) were chosen to describe the crumb grain.

### 2.8. Statistical Analysis

All measurements were performed at least in triplicate. The confidence interval was calculated and the results were checked using the Grubbs-test (outlier-test) with a significance level of α = 0.05. Pearson correlation coefficient (*r*) and *p*-value were used to show correlations and their significance using the Basic Statistics package of the software MINITAB version 15 (MINITAB Ltd., Coventry, UK). Differences of *p* < 0.05 were considered significant. The correlation coefficient is classified in different levels of correlation: perfect (|*r*| = 1.0), strong (0.80 ≤ |*r*| ≤ 1.0), moderate (0.50 ≤ |*r*|≤ 0.80), weak (0.10 ≤ |*r*| ≤ 0.50), and very weak (almost none) correlation (|*r*| ≤ 0.10).

## 3. Results and Discussion

### 3.1. Starch Granule Morphology

**Figure 1:**
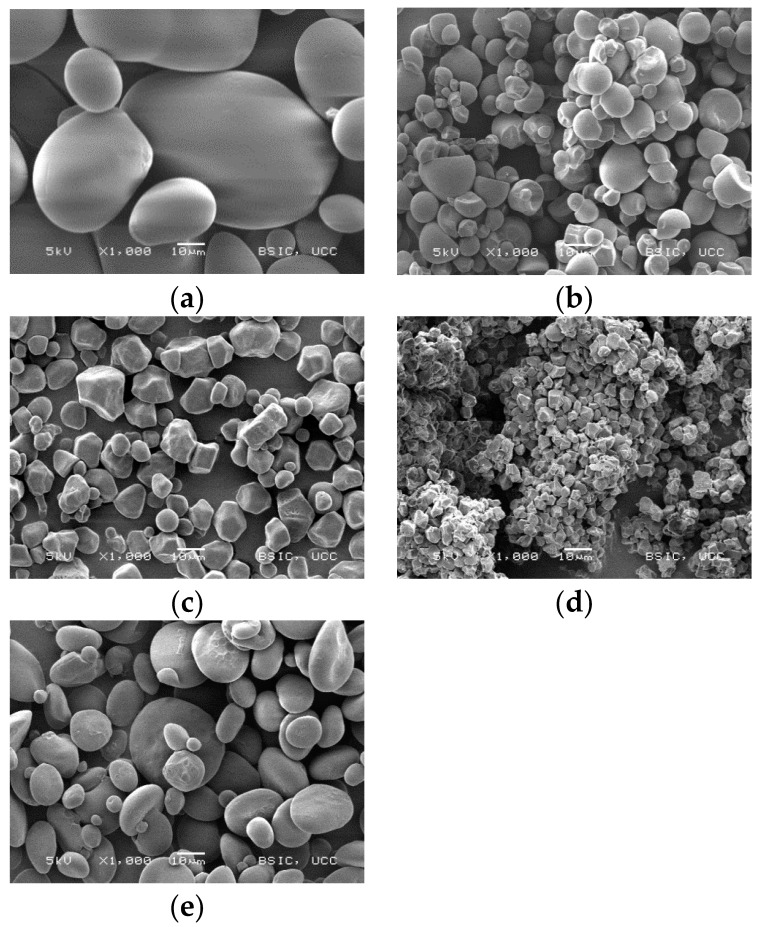
Micrographs of (**a**) potato starch, (**b**) tapioca starch, (**c**) corn starch, (**d**) rice starch, (**e**) wheat starch (Magnification 1000×).

SEM was used to compare the microstructure of the different starches. Different granule sizes (A and B granules), shapes and the mix of different size granules in the starch have an influence on the rheology and the functional and structural properties of starch based foods [14]. The morphological characterisations are depicted in Figure 1.

**Figure 2:**
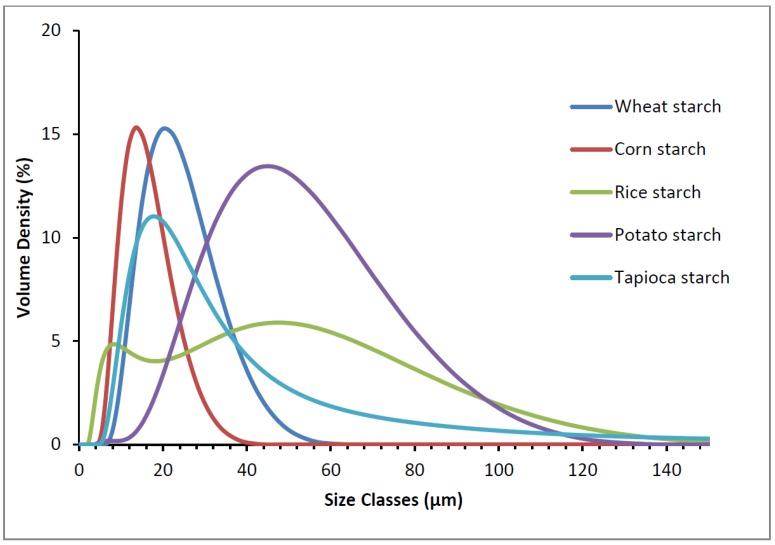
Granule size distributions of the different starches.

The granular size of the different starches was determined by laser diffraction using a dry feed cell. The results are shown in Figure 2.

The comparison of the Sauter mean diameter (d_32_) showed the following order, starting with the biggest one: potato starch (36.7 μm), gluten-free wheat starch (18.9 μm), tapioca starch (18.1 μm), corn starch (12.9 μm), and rice starch (12.3 μm). However, the size distribution showed that rice starch had high values, similar to potato starch. This is due to the agglomerated state of rice starch, which leads to a detection of big granules (Figure 1). The micrographs in Figure 1 reveal that the potato starch has big and small granules, referred to as A and B granules, respectively. Gluten-free wheat starch also showed big and small granules. For the tapioca starch granules, agglomerated granules were found.

### 3.2. Chemical Characterisation of the Starches

The results of the chemical characterisation of potato, tapioca, corn, rice, and gluten-free wheat starch are shown in Table 1.

The cereal starches (wheat, corn, and rice starch) contained a lower moisture level compared to the tuber starches (potato and tapioca starch). The total starch contents were, except for tapioca starch and corn starch, significantly different. From the starches analysed, gluten-free wheat starch showed the highest, and rice starch the lowest, total starch content. As expected, for the amylose and amylopectin contents significant differences between the starches were found. The potato starch had the highest amylose content (54.2%), while gluten-free wheat starch had the lowest (25.1%). Properties such as susceptibility to enzymatic hydrolysis and the gelling and pasting behaviour can be related to the amylose content. Furthermore, a higher content of damaged starch was found for the cereal starches. These differences were also detected by Schirmer *et al*. [14]. The higher content of damaged starch reflects the effect of milling, which is involved in the extraction process of rice and wheat starch. Corn starch undergoes wet milling which is a softer process leading to a lower starch damage content. Instead of milling, a process of rasping and washing (lixiviation) is applied for the extraction of starch from root and tubers [15]. The lipid content of the starches was also analysed. Low but significantly different values were determined. In the cereal starches, values between 0.26% and 0.71% were found, while in potato and tapioca starch no lipids were detectable. In general, the lipid content in starch is low and depends on the source of origin. It has been reported that the cereal starches have higher lipid contents than root and tuber starches [14]. However, even though the lipids belong to the minor components in starch, they have significant influence on the gelatinisation properties and on bread making properties. Lipids in starch occur, mainly due to their structure (linear), in complexes within the helical amylose or within the long branch-chains of amylopectin, or form complexes with amylose during heating [16]. It can be assumed that the difference in the lipid contents is influenced by the source of the starch and its internal structure. This assumption is supported by Lindeboom *et al*. [17], who analysed the biochemical and physiochemical aspects of starch granule size. The presence of lipids in starch is important in bread making due to the positive properties imparted during the baking process and the storage [18]. The effect of lipids on the baking process has been mainly reported for flours, where a higher lipid content and interactions with proteins, compared to starches occur [19]. These effects are also important in gluten-free baking. The main lipids in starch are polar lipids. Polar lipids can act as surface active components, which are known to stabilize liquid films at gas-liquid interfaces. This can help to maintain the integrity of gas cells during mixing and baking [18]. Hence, it could help gas retention as well as the expansion of gas cells/bubbles during proofing. During baking, the better cell wall stability leads to greater retention of evaporated water and CO_2_ produced by the fermentation process. This retention leads to a larger loaf volume and a finer crumb texture [19]. This explains the significantly higher loaf volumes of wheat and corn starch compared to potato starch breads.

Alpha-amylase is an important enzyme occurring in different starch sources [20]. It is an endogenous amylase, which is able to cleave α-1,4-glycosidic bonds present in the amylose or amylopectin chain. The end products of α-amylase action are oligosaccharides. However, no α-amylase activity was detected. In contrast to the α-amylase, beta-amylase is an exogenous amylase, which cleaves exclusively α-1,4-glycosidic bonds from the non-reducing end of the chain. The beta-amylase acts on the external glucose residues of amylose or amylopectin and only maltose and some glucose units are generated [20]. The significantly highest β-amylase activity was found in rice starch, it was more than seven times higher than the remaining starches. Amylases have an influence on baked breads, due to the production of maltose and glucose, which leads to improvement in colour as a result of caramelisation and Maillard reactions. Furthermore, these monosaccharides are fermented by yeast into alcohol and carbon dioxide, which causes rising of the bread [20].

### 3.3. Pasting Properties

**Figure 3:**
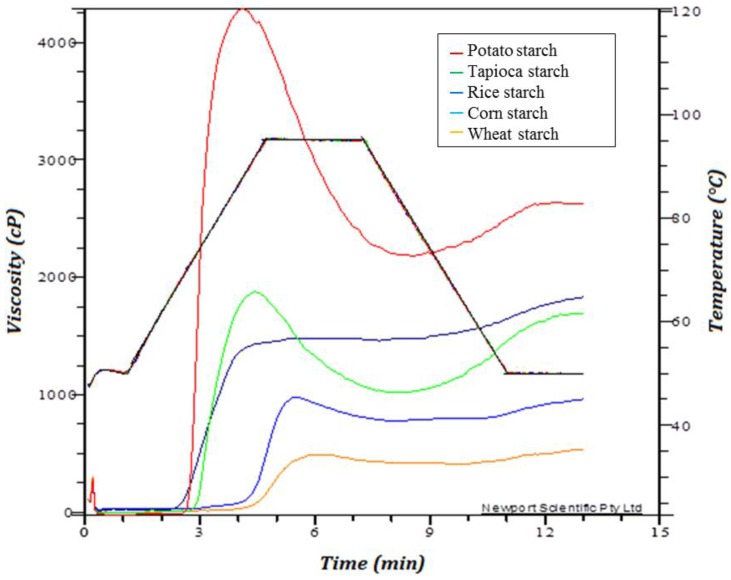
Pasting profiles of the different starches: Potato starch, tapioca starch, rice starch, corn starch and wheat starch.

The pasting properties of the different starches were determined with the rapid visco analyser (RVA), an instrument that measures the viscosity (cP) of a sample while a specific temperature profile is applied. Figure 3 illustrates representative RVA pasting patterns for the different starches.

The differences in pasting behaviour between the cereal and root/tuber starches are influenced by the different ratios of amylose and amylopectin in starch, which cause different degrees of gelatinisation. Starch gels are an amylose network, where swollen granules are immersed [16]. Furthermore, the lipid content of starch has an influence on pasting. The results obtained in this work showed that the cereal starches with the higher lipid contents had lower gelation viscosities and started to gelatinise later than the root and tuber starches. Based on these findings, the authors hypothesize a correlation between increasing lipid content and increasing gelatinisation temperature. This hypothesis is in agreement with literature, which reported that lipids increased the gelatinisation temperature, thereby retarding granule swelling and preventing the leaching of amylose during gelatinization [21,22]. However, the high gelation viscosity of potato starch is believed not only to be related to the absence of lipids. Schirmer *et al*. [14] demonstrated that potato starches have a high content of phosphate monoesters. The authors further stated that these are covalently bound to the amylose and amylopectin fraction, and that they induce a greater granule swelling causing a higher peak viscosity. Furthermore, the difference in pasting behaviour could be due to the granule size [14]. Sánchez *et al*. [23] stated that native starches with large granules, e.g., potato starch, display a unique swelling capability and form highly viscous pastes. However, the peak viscosity (PV) was positively correlated with the amylose content (|*r*| 0.94). In general, it had been found that large granules (e.g., potato starch) have a greater swelling capacity and therefore form highly viscous pastes [23] and are correlated with a greater breakdown of a viscosity curve. The granule size of the starches had an influence on this breakdown. Only gluten-free wheat starch did not correlate. This is due to the significantly higher damaged starch content, which was shown by Barrera *et al*. [24], to have an influence on the breakdown and setback. These authors stated that an increase in damaged starch would decrease the breakdown. Hence, for gluten-free wheat starch and rice starch, a very small breakdown was found.

In this study it was not possible to correlate the pasting behaviour of a starch to a single factor. The authors hypothesize that the pasting properties are dependent on many intrinsic and extrinsic factors instead of one single factor. This is in agreement with Abdel-Aal [2], who reported that, in general, the pasting properties of starch depend on the source and type of the starch, the amylose content, amylose/amylopectin ratio, molecular weight, percentage of starch damage, moisture content, lipid content, shear rate, temperature, and period of time during the measurement. The pasting properties could be linked to the firmness of the bread, as the potato starch showed higher viscosity values compared to corn and wheat, which showed a softer crumb. The lipid content also plays a role in the lowering of starch gelation and softening of bread crumb.

### 3.4. Model Bread Systems

#### 3.4.1. Bread Structure

**Figure 4:**
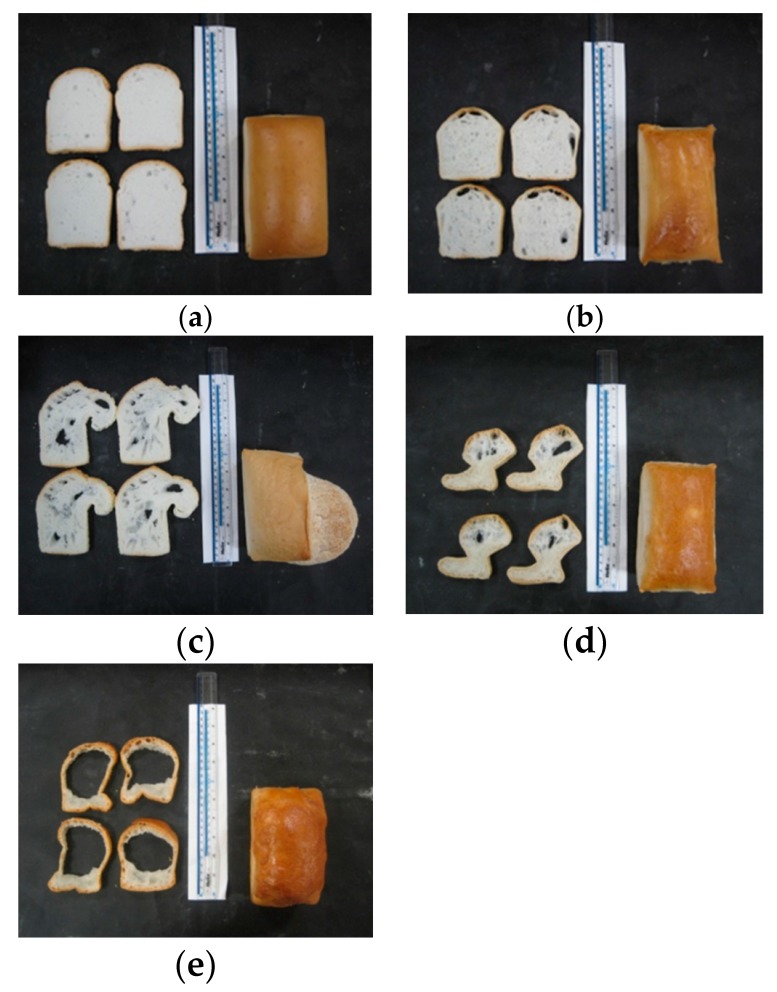
Images of the breads obtained with different starches: (**a**) wheat starch, (**b**) potato starch, (**c**) corn starch, (**d**) tapioca starch, and (**e**) rice starch.

Pictures of the model breads shown in Figure 4 illustrate that gluten-free wheat starch and potato starch gave the best crumb structure.

The analysis and correlation of starch characteristics with bread structure revealed that starches containing A and B granules resulted in the best bread structure (|*r*| 0.92). This is in agreement with Park *et al*. [25], who analysed the size distribution of gluten-free wheat starch granules in relation to crumb characteristics. These authors found that the breads with better crumb grain contained more A granules with larger sizes. Furthermore, it can be seen that during baking the dough in the corn-starch bread overflowed, which led to big holes in the crumb. As described earlier, higher lipid contents can hinder gelation. Thus, it is hypothesised that a lower lipid content causes a weaker stabilisation of the network interfaces, which leads to partial rupture of the network resulting in big holes. The big holes led to a greater specific volume (|*r*| 0.83), which in turn led to the high bake loss (|*r*| 0.89) of corn starch; due to the greater surface of the loaf, more water was able to evaporate. Rice starch and tapioca starch produced breads with an irregular structure and large holes. The rice and tapioca starch based breads could not be used for further study due to their poor structure. The SEM pictures, shown in Figure 1, revealed that rice starch and tapioca starch had small and agglomerated granules. Due to the fact that granule size correlates with bread structure, it is hypothesised that the agglomeration of granules could lead to a weak baking performance. Moreover, the inferior performance, particularly in rice starch, is also linked to the high damaged starch content and the high amylase activity. This may have caused a collapse of the interior of the bread, due to liquefying of the starch. The damaged starch can easily be cleaved by the high β-amylase activity [20], which results in a higher amount of maltose, some glucose, and loss of water holding capacity [26]. The high amount of maltose is fermented by the yeast during proofing which leads to increased formation of carbon dioxide and alcohol [20]. It is assumed that the carbon dioxide and the alcohol, which evaporates during baking, led to the inflation of the bread. The area of holes and the wall thickness were measured using a c-cell system (Table 2). For the area of holes, significant differences between the various starch based breads were found. These are most likely linked to the lipid contents in the starches as described earlier. The pictures (Figure 4) of the breads confirm the high value of the area of the holes in the corn starch based bread. No significant difference between the starch breads for the wall thickness and moisture content were found.

#### 3.4.2. Bread Texture

Crumb hardness is a very important quality characteristic of bakery products. The TPA results are shown in Table 2.

Potato starch bread showed significantly higher hardness values in comparison to the cereal starches. Correlation analysis showed that these results are linked to its high amylose content (|*r*| 0.92), which results in higher swelling power and the granule size. This is in agreement with literature [27,28], which states that during baking, amylose leaches from granules to the limited moisture system of bread dough and retrogrades when it is cooled and becomes inextricable. On the other hand, authors [29,30] proposed that amylose, based on its rapid rate of retrogradation, is responsible for setting the initial crumb structure, but is not involved in the staling process. A study by Hug-Iten *et al*. [6] revealed that a high amylopectin level is connected to the recrystallization after baking and can be correlated to crumb firming. Nevertheless Hug-Iten *et al*. [6] also mentioned that α-amylase could prevent the amylopectin recrystallization which would hinder bread firming. Moreover, a recent study on gluten-free baking by Mäkinen *et al*. [31] revealed that α-amylase has indeed a positive influence on the specific volume and on the crumb structure. However, in the current study, as stated above little α-amylase activity was found in the starches (data not shown).

It is reasonable to assume that the amylose/amylopectin ratio and the related amylose crystallisation and amylopectin retrogradation of a starch have also have a major influence on the hardness of baked bread, alongside granule size (|*r*| 1.00) and swelling power (|*r*| 0.92) and lipid content (|*r*| −0.71). Potato starch contained the highest amylose content (Table 1), which resulted in the highest hardness value. On the other hand, gluten-free wheat starch contained the highest amylopectin content, which resulted in the lowest hardness of the bread crumb (Table 2). This result leads to the conclusion that amylose crystallisation, in general, has a higher impact on bread hardening than amylopectin. This effect is probably based on the reason that amylose crystallises over a period of minutes to hours, while amylopectin retrogrades over hours or days [32,33]. The lipid content must also be considered when discussing the hardness/firmness/staling of bread. It is assumed that the lipid content lowers the hardness of the bread crumb by retarding the staling process. This assumption is supported by findings of Copeland *et al*. [16] and Keetels *et al*. [34] who mentioned that the developed lipid-amylose complexes retarded the retrogradation of amylose and the recrystallization of amylopectin, respectively. Additional effects on the bread structure and texture could be caused by starch-hydrocolloid interactions. HPMC is reported to delay bread staling and affect the pasting and rheological properties of starch [35]. Such influences are reported to be dependent on the specific starch-hydrocolloid interactions [36]. In this study, the water level was kept constant, to focus on the impact of starch. Due to this, the addition of HPMC could have restricted the pasting of starch, by limiting the available water for the pasting.

## 4. Conclusions

This study was conducted to investigate the impact of starches on a simple gluten-free bread system. It was observed that gluten-free wheat starch and potato starch performed better compared to the other starches in this study, in terms of bread loaf volume and crumb structure. It was found that the starches had a significant impact on the gluten-free model breads.

Correlation analysis revealed that the granule size of the starches has the highest impact on bread texture and structure. It correlated with the bake loss (|*r*| −0.88), specific volume (|*r*| −0.93), crumb moisture (|*r*| 0.99), and the staling rate (|*r*| 1.00). For the baked bread analyses, the rice starch and tapioca starch were excluded due to their lack of a bread structure. It is suggested that the high β-amylase activity and the high damaged starch content in rice starch lead to this weak performance. Although the results of the characterization of potato and wheat starches showed no similarity (except morphology) between them, the resulting bread structure was very similar. Overall, this study showed in a model bread system that gluten-free wheat starch is the best option for the production of gluten-free bread followed by potato starch in terms of volume and bread structure. This study contributes to the knowledge of gluten-free baking by highlighting the differences of various starches in a simple model bread system. The correlation between granule size and baking characteristics further supports the idea that larger granules are better suited to gluten-free bread production. Although the morphology of the starches has a major impact on the final product, the differences in the composition of the starches should not be neglected. Therefore, further research on the effect of starches from the same source, but of different composition could give further insights into the importance of starch source or composition. In addition, further research on interactions between different components and their behaviour in a model bread system could provide a deeper understanding of gluten free systems and help to gain a fundamental understanding of how wheat flour can be replaced by gluten-free ingredients.

## Figures and Tables

**Table 1:** Overview of the different starch compositions. Results are shown as the mean values ± confidence interval of 3 replicates.

Starch Sample	Moisture (%)	Total Starch (db) (%)	Amylose (%)	Damaged Starch (db) (%)	Protein (db) (%)	Lipids (db) (%)	Beta-Amylase (U/g)
Potato	18.0 ± 0.1 ^a^	92.2 ± 2.1 ^a^	54.2 ± 0.6 ^a^	1.0 ± 0.1 ^a^	0.02 ± 0.03 ^a^	n.d.	0.18 ± 0.01 ^b^
Tapioca	13.7 ± 0.0 ^b^	94.2 ± 0.1 ^b^	36.0 ± 0.3 ^b^	0.7 ± 0.0 ^b^	0.03 ± 0.04 ^a,b^	n.d.	0.25 ± 0.01 ^b^
Corn	12.2 ± 0.1 ^c^	92.5 ± 0.9 ^b^	28.9 ± 0.2 ^c^	1.0 ± 0.1 ^a^	0.04 ± 0.05 ^a,b^	0.26 ± 0.01 ^a^	0.32 ± 0.0 ^b^
Rice	12.5 ± 0.1 ^d^	83.4 ± 2.5 ^a^	46.4 ± 0.4 ^d^	7.4 ± 0.2 ^c^	0.04 ± 0.06 ^b^	0.71 ± 0.02 ^b^	2.33 ± 0.15 ^a^
Wheat	12.8 ± 0.2 ^e^	97.4 ± 5.7 ^b^	25.1 ± 0.3 ^e^	2.5 ± 0.2 ^d^	0.10 ± 0.14 ^c^	0.50 ± 0.01 ^c^	0.33 ± 0.0 ^b^

Means across a single column with different superscripts are significantly different (*p* < 0.05); Means across a single column without superscripts are not significantly different (*p* > 0.05).

**Table 2:** Overview of the baking results: Results are shown as the mean values ± confidence interval of at least nine replicates.

Starch	Bake Loss (%)	Specific Volume (mL/g)	Crumb Moisture (%)	C-Cell		Texture Profile Analysis		
				Area of Holes (%)	Wall Thickness (mm)	Day of Analysis	Hardness (N)	Cohesiveness (-)
Potato starch	18.3 ± 2.7 ^a^	3.3 ± 0.1 ^a^	49.2 ± 1.8 ^a^	4.1 ± 1.6 ^a^	0.5 ± 0.1 ^a^	Day 0	4.2 ± 0.5 ^a^	0.71 ± 0.02 ^a^
						Day 2	24.2 ± 2.0 ^a^	0.55 ± 0.05 ^a^
						Day 5	28.8 ± 2.0 ^a^	0.53 ± 0.04 ^a^
Corn starch	20.9 ± 3.6 ^b^	5.0 ± 0.3 ^b^	48.2 ± 0.3 ^a^	12.0 ± 1.0 ^b^	0.5 ± 0.1 ^a^	Day 0	3.2 ± 0.6 ^b^	0.69 ± 0.02 ^a^
						Day 2	17.7 ± 4.4 ^b^	0.59 ± 0.06 ^a^
						Day 5	20.7 ± 3.4 ^b^	0.54 ± 0.07 ^a^
Wheat starch	19.1 ± 2.6 ^a,b^	4.0 ± 0.1 ^c^	48.3 ± 0.2 ^a^	2.4 ± 1.7 ^c^	0.5 ± 0.1 ^a^	Day 0	3.0 ± 0.4 ^b^	0.75 ± 0.01 ^a^
						Day 2	14.9 ± 0.8 ^b^	0.67 ± 0.05 ^b^
						Day 5	22.5 ± 0.9 ^b^	0.57 ± 0.05 ^a^

Means across a single column with different superscripts are significantly different (*p* < 0.05).
